# Effects of bumetanide on neurobehavioral function in children and adolescents with autism spectrum disorders

**DOI:** 10.1038/tp.2017.10

**Published:** 2017-03-14

**Authors:** E Lemonnier, N Villeneuve, S Sonie, S Serret, A Rosier, M Roue, P Brosset, M Viellard, D Bernoux, S Rondeau, S Thummler, D Ravel, Y Ben-Ari

**Affiliations:** 1 CHU hoputal Le Cluzeau, CHU Limoges, Limoges, France; 2 CHU hoputal Le Cluzeau, Hôpital Sainte Marguerite, Marseille, France; 3 CHU hoputal Le Cluzeau, CHU le Vinatier, Bron, France; 4 CHU hoputal Le Cluzeau, CHU Lenval, Nice, France; 5 CHU hoputal Le Cluzeau, CHU Le Rouvray, Sotteville les Rouen, France; 6 CHU hoputal Le Cluzeau, CHRU Brest, Brest, France; 7 CHU hoputal Le Cluzeau, Neurochlore Research Team, Marseille, France; 8 CHU hoputal Le Cluzeau, Neurochlore Research Team, C/O INMED, INSERM Unité 901, Aix Marseille Université, Marseille, France

## Abstract

In animal models of autism spectrum disorder (ASD), the NKCC1 chloride-importer inhibitor bumetanide restores physiological (Cl^−^)_i_ levels, enhances GABAergic inhibition and attenuates electrical and behavioral symptoms of ASD. In an earlier phase 2 trial; bumetanide reduced the severity of ASD in children and adolescents (3–11 years old). Here we report the results of a multicenter phase 2B study primarily to assess dose/response and safety effects of bumetanide. Efficacy outcome measures included the Childhood Autism Rating Scale (CARS), the Social Responsive Scale (SRS) and the Clinical Global Impressions (CGI) Improvement scale (CGI-I). Eighty-eight patients with ASD spanning across the entire pediatric population (2–18 years old) were subdivided in four age groups and randomized to receive bumetanide (0.5, 1.0 or 2.0 mg twice daily) or placebo for 3 months. The mean CARS value was significantly improved in the completers group (*P*: 0.015). Also, 23 treated children had more than a six-point improvement in the CARS compared with only one placebo-treated individual. Bumetanide significantly improved CGI (*P*: 0.0043) and the SRS score by more than 10 points (*P*: 0.02). The most frequent adverse events were hypokalemia, increased urine elimination, loss of appetite, dehydration and asthenia. Hypokalemia occurred mainly at the beginning of the treatment at 1.0 and 2.0 mg twice-daily doses and improved gradually with oral potassium supplements. The frequency and incidence of adverse event were directly correlated with the dose of bumetanide. Therefore, bumetanide improves the core symptoms of ASD and presents a favorable benefit/risk ratio particularly at 1.0 mg twice daily.

## Introduction

The regulation of the intracellular neuronal chloride ((Cl^−^)_i_) levels determines the efficacy of GABAergic inhibition: high levels can reverse the polarity of GABA actions from inhibition to excitation. In contrast to immature neurons that have high (Cl^−^)_i_ levels,^1, 2^ adult ones have usually low (Cl^−^)_i_ levels and inhibitory actions of GABA. However, high (Cl^−^)_i_ levels and excitatory actions of GABA are produced by a wide range of disorders and insults including seizures, brain trauma, spinal cord lesions, cerebrovascular infarcts or chronic pain.^1, 2, 3, 4, 5, 6, 7, 8, 9^ As GABAergic networks have essential roles in the generation of behaviorally relevant oscillations,^1, 2, 10, 11^ a polarity shift of the actions of GABA will impact sensory and integrative properties of the brain and exert major deleterious effects. These observations have raised considerable interest in the development of pharmacological treatments that restore physiological (Cl^−^)_i_ levels and GABAergic inhibition in pathological conditions.

In experimental conditions, the high affinity specific NKCC1 chloride-importer inhibitor bumetanide reduces (Cl^−^)_i_ levels, restores GABAergic inhibition and attenuates the severity of electrical or behavioral manifestations in many pathological conditions.^1, 2, 3, 4, 5, 6, 7, 8, 9, 12, 13, 14^ Used since 1975 in adults and since 1986 in children to treat hypertension, bronchopulmonary dysplasia, nephritic syndromes and congestive heart failure, bumetanide has limited side effects restricted primarily to hypokalemia.^15, 16^ Bumetanide is therefore a good candidate to test clinically.

Autism spectrum disorders (ASD) are associated with social disability, communication impairment, repetitive behaviors and restricted interests (International Classification of Diseases-10).^17^ Genetic mutations,^18, 19^ preterm delivery, intrauterine inflammation, drug exposure during pregnancy and delivery complications^20, 21, 22^ contribute to the pathogenesis of ASD. There is no Federal Drug Administration or European Medicine Agency (EMA) approved treatment for the core symptoms of ASDs. Two drugs granted authorization to treat autism-related irritability: risperidone (5–16 years old) and aripiprazole (6–17 years old). Risperidone's beneficial effect on aggression is not accompanied by a reduction in core ASD symptoms.^23^ Both risperidone and aripiprazole have important side effects including sedation, vomiting, extrapyramidal syndromes, increased appetite, drowsiness, drooling and body weight gain.^23, 24, 25^ Clinical trials based on the mGluR5 antagonist concept to treat Fragile X^26, 27^ have provided contradictory results.^28, 29^ A pilot study suggests that the GABA-B agonist might improve social function and behavior in patients with Fragile X.^30^ Promising short lasting results have been obtained with intranasal oxytocin^31, 32^ that might also be mediated by regulation of (Cl^−^)_i_ levels.^33^ It is therefore important to develop novel therapeutic avenues.

Cortical neurons have high (Cl^−^)_i_ and excitatory actions of GABA in the valproate animal model of ASD and Fragile X mice, and bumetanide attenuates electrical and behavioral features of autism in both models.^5, 12, 34, 35, 36^ Relying on these observations, we have performed a series of pilot and large clinical trials to test the efficacy of bumetanide to treat ASD. Following promising open-label pilot trials,^37, 38^ we reported positive results in a single-site double-blind study in 54 children as assessed by Childhood Autism Rating Scale (CARS) and the Clinical Global Impression (CGI).^37^ Bumetanide also improved visual recognition of emotive figures in adolescents with ASD.^39^ With functional magnetic resonance imaging, the fusiform face area and the inferior occipital gyrus involved in perception of emotional faces were activated by emotive and fear figures after but not before bumetanide treatment.^39^ Other beneficial effects of bumetanide have been observed in pilot reports in children with ASD or with Fragile X syndrome^40, 41^ and a double-blind clinical trial.^42^

We report here the results of a multicenter phase II dose-ranging study conducted according to a pediatric investigation plan approved by the EMA to determine the optimum bumetanide dose in ASD children and adolescents aged 2–18 years. To achieve these aims, the study population was divided into four subgroups receiving three doses of bumetanide (0.5, 1.0, 2.0 mg twice a day) or placebo. The distribution of age was the same in the four groups (2–4, 5–9, 10–13, 14–18 years old). The results of this trial confirm and extend our earlier observation that bumetanide improves the symptoms of ASD and that too in the full age range targeted.

## Materials and methods

### Design

We performed a double-blind, randomized, placebo-controlled, multisite dose-ranging study to assess the efficacy, safety, pharmacokinetics and the optimal dose of bumetanide in children and adolescents with ASD. The study was conducted in accordance with the Declaration of Helsinki and good clinical practice and was approved by the Committee of Persons Protection (Sud Mediterranée V), the French Health Agency (ANSM), clinical registration number (NCT01078714). Neuroclin02 study is part of the pediatric investigation plan approved by EMA (EMEA-001303-PIP01-12). The scope, the design and the analyses of this study were discussed with EMA/Pediatric Committee (PDCO). Parents provided written informed consent, and patients provided written informed assent when possible. More details and Supplementary Information accompany this paper online.

### Trial procedures

The patients were enrolled in six French specialized centers (hospitals of Brest, Limoges, Rouen, Nice, Lyon and Marseilles) from January 2014 to March 2015. They first attended a screening and baseline visit within 6 weeks; following which eligible patients were assigned (1:1:1:1) to either bumetanide (0.5, 1.0, 2.0 mg, twice a day) or placebo according to a computer-generated age-stratified randomization schedule prepared by Amatsi group (Saint-Gély du fesc, France). Children with epilepsies were excluded as well as psychotropic medications (including antipsychotic, psychostimulant, antidepressant, anxiolytics, mood stabilizers and neuroleptic agents) that had to be discontinued at least 4 weeks before entering the trial. This was essential both to determine specifically the effects of bumetanide and to prevent possible unknown potential interactions. No additional medication was allowed with the notable exception of melatonin—to treat sleep disorders. Aminoglycosides were prohibited because of possible ototoxic effects. All recruited patients were outpatients treated in centers specialized for pediatric autism (Centre de Ressources Autisme). They were not allowed to start concurrent psychotherapy, social skills training or behavioral interventions. The EMA-approved protocol did not consider global intelligence as an eligibility criterion as in similar trials in France and taking into account the difficulty of intelligence quotient tests in small children. Most of the patients between 2 and 11 of age were at the nursery or the primary school. The Centre de Ressources Autisme centers are staffed by a multidisciplinary team specialized and experienced in the autistic syndrome, implementing actions such as early diagnosis, research, assistance, support, information, training, consulting and expertise with families and community health professionals.

To enter the study, patients had to fulfill diagnosis criteria of Childhood Autism (F84.0) or Asperger's Syndrome (F84.5) according to International Classification of Diseases-10; and Criteria for Autism Diagnostic Observation Schedule—Generic (ADOS G) and Autism Diagnostic Interview-Revised (ADI-R). They had to have a CARS total score >34 points and a weight ⩾11 kg. Serious, unstable illnesses including, gastroenterological, respiratory, cardiovascular (QT interval lengthening), endocrinology, immunologic or hematologic disease; renal or hepatic dysfunction and neurological disorders such as seizures and microcephaly were not allowed.

The study consisted of nine visits over a period of 19/20 weeks as follows: visit 1 (week-3, screening), visit 2 (day 0, baseline), visit 3 (day 7), visit 4 (day 14), visit 5 (day 21), visit 6 (day 28), visit 7 (end of month 2), visit 8 (end of month 3) and visit 9 (end of month 4, follow-up). Bumetanide was packaged and labeled by an independent Contract Research Organization. The placebo solution was visually indistinguishable from bumetanide and packaged in the same way to that of bumetanide. Both the placebo and bumetanide were labeled (pre-printed, indistinguishable) with the randomization number and site number. Each patient was seen and assessed by two clinicians who were unaware of the treatment assignment. A pediatrician who was responsible for the management of the patient's condition, including safety assessments, inter-current illnesses, tolerability and prescription of medication and follow-up on the other protocol-related procedures and a child psychiatrist who assessed the response to treatment before the beginning of the treatment and on day 90. The clinicians had no contact with each other before the lock of the database. The two clinicians were based on different clinical sites. Blood samples for pharmacokinetic analysis were drawn from all the patients (bumetanide and placebo) and sent to a central laboratory for analysis. At each visit weight, vital signs, blood and urine, and laboratory tests were assessed including electrolytes, calcemia, uric acid, urea, creatinine, glucose, hematocrit, protein, alkaline phosphatase, ALAT, ASAT, gamma GT and renin and aldosterone activities (only at screening visit and day 90), as well as electrocardiogram and renal ultrasound measures. Adverse events were documented according to severity, duration, attribution and outcome.

### Power calculation of the sample size and rationale for age subgroups

A sample size of 76 patients (19 in each treatment group) was required for 90% power to detect a mean difference of 4 points in change from baseline to day 90 in the CARS score between active and placebo treatments using analysis of variance with 5% significance. This calculation was based on an assumption that the standard deviation for change in CARS was 4.5. For a sample of 80 evaluable patients, the power was 92.5%.

EMA imposed the age range from 2 to 18. The rationale for age subgroups was to recruit the same number of children (2–8 years) and adolescents (9–18 years). To ensure the same age distribution in each treated groups, these two groups were divided in two subgroups 2–4 and 5–8 and 9–13 and 14–18. The mean age in the four groups was the same: 7.8±4.1 (0.5 mg), 7.9±4.6 (1.0 mg), 8.4±4.6 (2.0 mg) and 8.8±4.5 (placebo).

A Data Safety Monitoring Board including a pediatric neurologist, a cardiologist, nephrologist and an ASD diagnosis expert oversaw the trial. A quality assurance and quality control system were implemented to ensure that the trial was conducted and data generated, documented and reported in compliance with the protocol and the regulatory requirements. Following the completion of the study, subjects were offered bumetanide for 6 months as compassionate use. The sponsor had no responsibility for the collection and analysis of the data that were entirely performed under the auspices of an independent Contract Research Organization (Simbec-Orion, Slough, UK).

### Medication schedule

**Figure 1:**
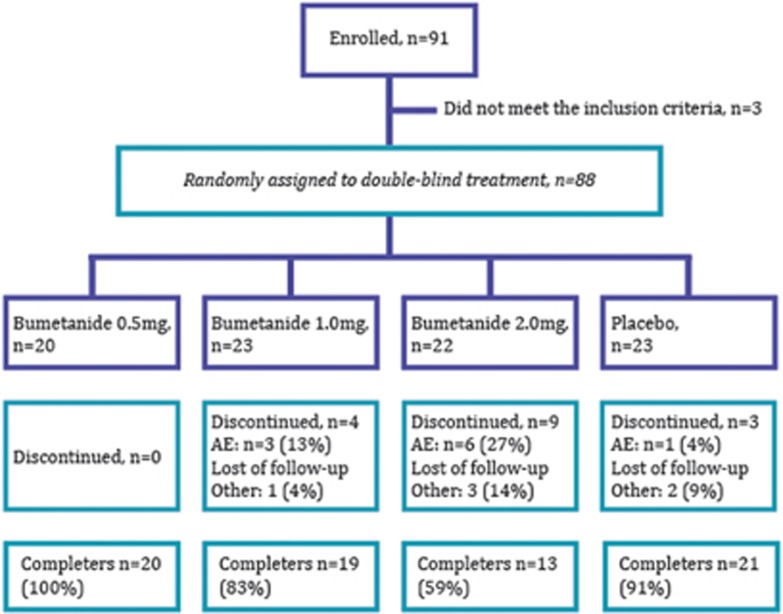
Patient flow in each treatment groups throughout the study. The enrolled and the included patients are noted followed by the four groups who received 0.5, 1.0 and 2 mg twice daily or placebo. Then the number of patients discontinuing the trial because of an adverse event (AE) or loss to follow-up is indicated, followed by the number of patients who completed the trial for each subgroup. One of the three discontinued patients in the placebo group withdrew after the visit on day 90 and was considered as a completer.

Patients were assigned to receive a newly developed pediatric liquid bumetanide formulation (0.5 mg ml^−1^) or a placebo identical in color and taste manufactured by Amatsi group. They received 0.5 1.0 or 2.0 mg, twice daily or placebo for a 3-month double-blind treatment period (Figure 1). For patients with a body weight of ⩾25 kg, bumetanide was given at 0.5, 1.0 or 2.0 mg b.i.d. or placebo. For patients with a body weight of <25 kg, the bumetanide dose was calculated on a body weight basis (doses of 0.02, 0.04 or 0.08 mg kg^−1^ b.i.d.). To have the same age distribution in each treatment group, assignment to dose 1, 2, 3 or placebo relied on a computer-generated randomization schedule stratified by age subgroups (2–4, 5–8, 9–13 and 14–18 years). Bumetanide liquid formulation was administered by means of a graduated dosing syringe before breakfast and at the end of the afternoon with a minimum of 6 h between doses.

### Baseline assessment and outcome measures

Each patient was seen during the screening period and on day 90 by a child psychiatrist who assessed the response to treatments relying on CARS as a primary end point. Clinical Global Impressions Improvement scale (CGI-I), Social Responsive Scale (SRS) were analyzed as secondary end point. CARS has been selected by the EMA in their recent (2016) guidelines for evaluation of ASD core symptoms (Guidelines on the clinical development of medicinal products for the treatment of ASD, 25 December 2015).

### Pharmacokinetics

A population pharmacokinetic analysis was performed on the patients treated with bumetanide for which complete treatment information was available. Bumetanide concentrations were measured at two visits (day 1 and day 90 of treatment). To optimize the blood-sampling scheme, the patients were stratified by two levels of age, 2–8 and 9–18 years. To prevent pain and stress related to blood sampling in children, a pharmacokinetic population approach requiring fewer blood samples per patient was conducted. The objective was to compare the pharmacokinetic profile of Bumetanide in pediatric population with that reported in adults (Supplementary Figure 1 and more details in Supplementary Material).

### Statistical analysis

All efficacy analyses were performed in the full analysis set (FAS) defined as all randomized patients. The primary end point was the change from screening to D90 of the total CARS score. Secondary efficacy end points included CGI and CGI-I at D90; change from screening in SRS at D90.

#### Pre-specified analyses

Owing to the low number of patients and the fact that the type of distribution of the change (day 90/screening) for CARS and SRS was unknown, we applied nonparametric tests. The analysis of the primary end point was performed using the Kruskal–Wallis test with Steel–Dwass adjusted pair-wise comparisons in the FAS assuming a heterogeneous distribution of scores. Missing data at D90 were imputed as no change (last observed carried forward (LOCF) approach). The same model was repeated for completers, that is, when a CARS value at screening and at day 90 was available. The same analyses were performed for all secondary efficacy end points.

#### *Post hoc* analyses

In a *post hoc* analysis, we found that the change (day 90/screening) for CARS and SRS followed a normal distribution (Supplementary Figures 4 and 5). We therefore performed a *post hoc* analysis using a general linear model with screening value as covariate in the FAS population. To take into account the multiplicity, Dunnett's multiple comparison procedure was used. For missing data at D90, the LOCF imputation method was implemented (Supplementary Tables 3–5). A sensitivity analysis to missing data was performed using the multiple imputation method (Supplementary Tables 4 and 6). A responder's analysis was performed in the FAS with high responders defined as patients for whom the CARS reduction was six points or more between screening and day 90.

## Results

### Subjects, disposition and dosing

Eighty-eight out of the 91 children and adolescents, who were enrolled for the study, met the eligibility criteria (78 males and 10 females; Figure 1). Thirteen patients in the bumetanide groups discontinued before the end of the study because of adverse events including 4 moderate hypokalemia, 5 adverse events related to diuresis, 2 difficulties complying with the study protocol, 1 lost to follow-up and 1 error in diagnosis. In addition, two patients in the placebo group discontinued because of difficulties to comply with the study protocol (*n*=1), and concomitant psychotropic medications (*n*=1). Therefore, 73 out of the 88 patients completed the trial; patient disposition is shown in Figure 1.

### Demographics and initial diagnosis

At screening, the patients ranged in age from 2 to 17 years (mean±s.d.: 7.8±4.1, 7.9±4.2, 8.4±4.6 and 8.9±5.0 for bumetanide 0.5, 1.0 and 2.0 mg and placebo, respectively). The four groups were similar with respect to age range, number of patients included and clinical characteristics, including mean baseline scores on CARS and SRS scales (Supplementary Table 1).

### Pharmacokinetics

In keeping with earlier studies, we found a rapid absorption and short half-life (from about 40–90 min) of the bumetanide liquid formulation (Supplementary Figure 1). Because of the small size of subpopulations and the large age range, the stratification was analyzed in two age groups (2–8 and 9–18 years old). Exposures (AUCt) calculated from individual predictions from the model were 77.5, 180.9 and 249.9 ng h ml^−1^, in the 0.5, 1.0 and 2.0 bumetanide groups, respectively for children aged 2–8 years. It was of 47.3, 126.7 and 158.4, respectively in the 0.5, 1.0 and 2.0 mg bumetanide groups for patients aged 9–18 years (Table 1). These results confirm that the pharmacokinetic parameters of bumetanide 0.5 mg ml^−1^ solution in this pediatric population compares with those described in adults.^43, 44, 45, 46^

### Safety outcomes

Treatment emergent adverse events (TEAEs) are described in Table 2. The most frequent TEAEs included hypokalemia, diuresis and loss of appetite, dehydration and asthenia. They were all related to the inhibitory effects of bumetanide on NKCC2, the luminal Na-K-2Cl symporter, in the thick ascending limb of Henle's loop which modulates water and electrolyte homeostasis.^47, 48, 49^ The frequency and incidence of TEAEs were directly correlated with the dose of bumetanide administered. The greatest frequency of TEAEs was in the 2.0 mg bumetanide group (121) followed by 1.0 mg (118), 0.5 mg (76) and placebo (39). The small difference between groups 2.0 and 1.0 mg was due to the larger number of dropouts in the 2.0 mg group. All TEAEs resolved on discontinuation of treatment.

Four TEAEs were classified as serious. Three were considered related to bumetanide: in the 0.5 mg b.i.d. group, one patient was overdosed due to parent's inattentiveness; and in the 1.0 and 2.0 mg b.i.d. bumetanide groups, one patient had a moderate hypokalemia. Ten patients, 7 in the 2.0 mg group and 3 in the 1.0 mg group were withdrawn from the study. The rate of withdrawal was 0%, 17% and 43%, respectively in the 0.5, 1.0 and 2.0 mg b.i.d. bumetanide groups, as compared with 14% in the placebo group. The withdrawals from the study because of an adverse event were reported for three patients in the 1.0 mg b.i.d. group: anorexia and fatigue (*n*=1), change in behavior (*n*=1), moderate hypokalemia (*n*=1); and for six patients in the 2.0 mg b.i.d. group: moderate hypokalemia (*n*=3), anorexia and fatigue (*n*=2) and polyuria (*n*=1). Blood pressure and results of routine laboratory tests did not differ significantly between the bumetanide and placebo groups. No hearing loss was reported. Kidney ultrasound did not reveal any abnormalities due to the treatments. TEAEs were similar with earlier observations,^43, 50^ in adults receiving loop diuretics.

As expected, the most frequent TEAE observed in bumetanide-treated groups was hypokalemia: either mild (between 3.0 and 3.5 mm) or moderate (between 2.0 and 3.0 mm). Hypokalemia occurred shortly after the start of the trial and were detected at the first visit after the beginning of the treatment on day 7 in the 1.0 and 2.0 mg b.i.d. groups. All the patients experiencing hypokalemia were administered gradual potassium supplements and were advised to eat potassium-rich foods. These procedures permitted a progressive return to normal (Supplementary Figure 2). In addition, the hypokalemia occurred most frequently during the first week of treatment and was dose dependent occurring more frequently with the 1 or 2 mg dose than the 0.5 mg bumetanide dose (Supplementary Figure 3). There was no straightforward relationship between age and TEAEs. Frequency of moderate hypokalemia was comparable when patients are split into two age subgroups 2–18 and 9–18 years old (Supplementary Table 2). No patients with mild or moderate hypokalemia developed cardiac arrhythmia or neuromuscular complications. Hypokalemia was accompanied by hypochloremia but not by hyponatremia and rapidly improved with high-potassium food and oral potassium supplementation. Interestingly, in 19 patients who participated in the 6-month extension and who received bumetanide at 0.5 mg b.i.d. for the first week and then shifted to 1.0 mg b.i.d., moderate hypokalemia (2.9 mm) was observed in only one patient (5%) on day 180, suggesting that a gradual dose increase might reduce hypokalemia. Therefore, bumetanide-induced TEAEs in children and adolescents are consistent with earlier adults studies, and the medication is safe provided that electrolytic balance is monitored and controlled.^47, 48, 49^

### Primary outcomes

The synopsis of the trial aimed primarily at determining the most efficient doses in the entire pediatric population resulted in small subpopulations thereby precluding detailed analysis of the outcomes on a subpopulation basis. All efficacy data are summarized in Table 2.

#### CARS score

The mean initial CARS scores were similar across all treatment groups in the six clinical centers and were above the cut-off for severe autism as required by EMA (>34). In the FAS, the mean values for CARS across all bumetanide-treated groups decreased in 90 days more than in the placebo group. The Kruskal–Wallis test for any difference between treatment groups did not reach statistical significance (*P*-value=0.068) in the FAS.

The difference between bumetanide 1.0 mg versus placebo in the FAS population was not statistically significant with the *post hoc* general linear model (*P*=0.3966) but the difference between bumetanide 0.5 mg and placebo was statistically significant −3.06 (95% confidence interval=[−5.63; −0.49], *P*=0.015) (Supplementary Table 3). Similar dose-dependent differences between bumetanide and placebo were also found with the multiple imputation method (Supplementary Table 4).

**Figure 2:**
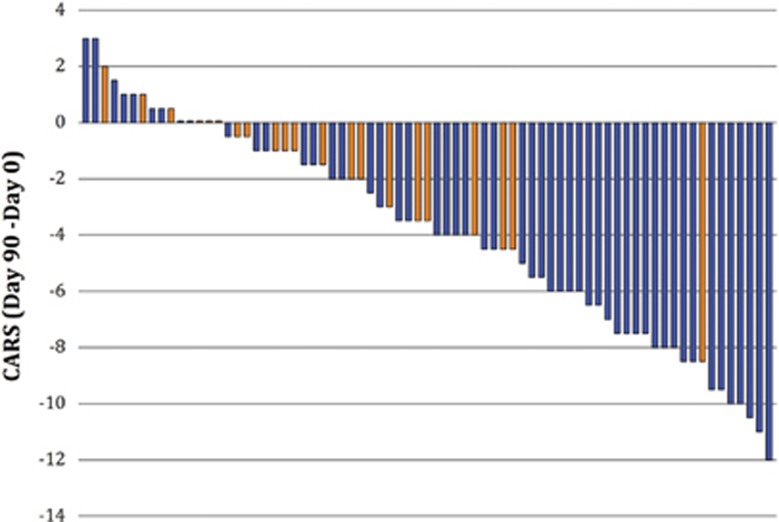
Change in the completers of the total CARS score from screening to day 90 after bumetanide (blue bars; *n*=52) and placebo (orange bars; *n*=21). All changes were calculated from the initial values for each individual participant at screening. Note a significant amelioration of the CARS scale after the treatment period (>4) is almost entirely restricted to the bumetanide-treated patients (only placebo). CARS, Childhood Autism Rating Scale.

For completers, the mean values decreased from screening to day 90 and the difference between all treatment groups was significant (Kruskal–Wallis *P*=0.015). The greatest mean change was in the bumetanide 2.0 mg group (−5.35±3.88) and the smallest in the placebo (−1.79±2.39; Steel–Dwass *P*=0.017; Table 2). The proportion of high responders in CARS scores differed significantly between bumetanide- and placebo-treated groups. Thus, 23 bumetanide-treated and only one placebo showed more than six-point reduction in CARS from screening to day 90 (bumetanide 0.5 mg, 50% *n*=10; 1.0 mg 22% *n*=5; 2.0 mg 36% *n*=8) and placebo (4% *n*=1; *P*=0.0057; Table 3). These differences are statistically significant (Table 3). This is also illustrated in Figure 2, where each bar represents individual CARS change in bumetanide (blue) and placebo (orange) completers. With one exception, no patient-receiving placebo had an improvement of more than five CARS points. CARS improvement was greater in the older patients (age 9–18, bumetanide 4.59±4.24 vs placebo 1.50±2.13) than young children (age 2–8, bumetanide 2.97±3.50 vs placebo 1.75±2.60). Therefore, bumetanide improves core ASD symptoms determined with the CARS scale.

#### SRS score

The difference between all treatment groups in reduction of global SRS score was statistically significant with the Kruskal–Wallis test (*P*=0.02; Table 2). The amelioration was greater than 10 points at the three doses tested with the highest reduction in the 2.0 mg bumetanide treatment group (−21.8±19.8 vs −1.55±20.38, respectively for bumetanide and placebo). Among the SRS subscales, statistically significant differences were observed in the subcategories social communication (*P*=0.039) and restricted interests and repetitive behavior (*P*=0.002; Table 4) but not in social cognition, social motivation or social awareness. With the *post hoc* general linear model and the LOCF method for handling missing data at D90, the difference between bumetanide and placebo was statistically significant with the 2.0 mg but not the 0.5 or 1 mg (Supplementary Table 5). With the *post hoc* general linear model and the multiple imputation method, the difference between bumetanide versus placebo in the FAS population was statistically significant with the 1 and 2 mg (*P*=0.036 and 0.008) but not the 0.5 mg population (*P*=0.261; Supplementary Table 6). Therefore, bumetanide attenuates in completers the global SRS score particularly on social communication and restricted interests.

#### CGI-I score

The CGI-I determined at the end of the trial (D90) was significantly different for total score between bumetanide and placebo groups (*P*=0.0043). Only one patient on placebo was much improved at day 90 compared with 17 bumetanide-treated patients (Table 2). No dose dependency was observed for the bumetanide-treated patients.

**Figure 3:**
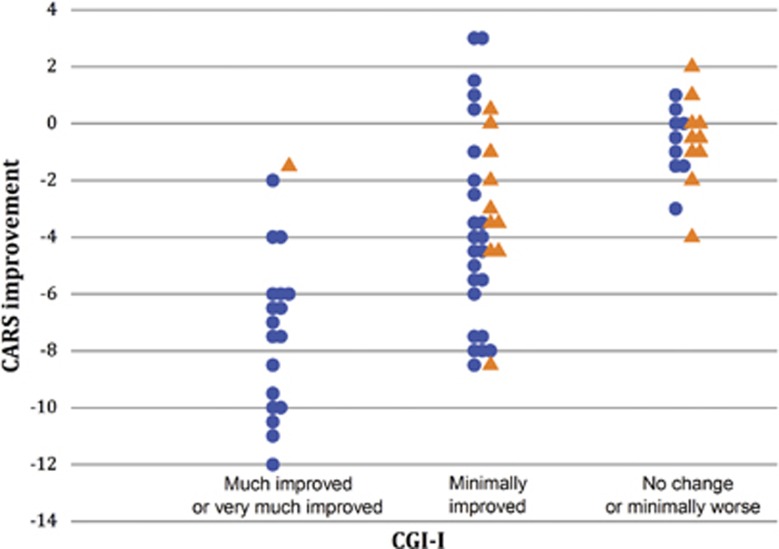
Attenuation in the CARS scale correlates with CGI attenuation. CARS scores and CGI-I values for completers are shown after the end of the trial (D90). Bumetanide treated are in blue circle (*N*=52) and Placebo in orange triangles (*N*=21). Note that the much- or very-much-improved children with CGI evaluation were almost entirely restricted to the bumetanide-treated group (only one placebo) and correlated with the most important CARS attenuation (ordinate). CARS, Childhood Autism Rating Scale; CGI-I, Clinical Global Impressions Improvement scale.

**Figure 4:**
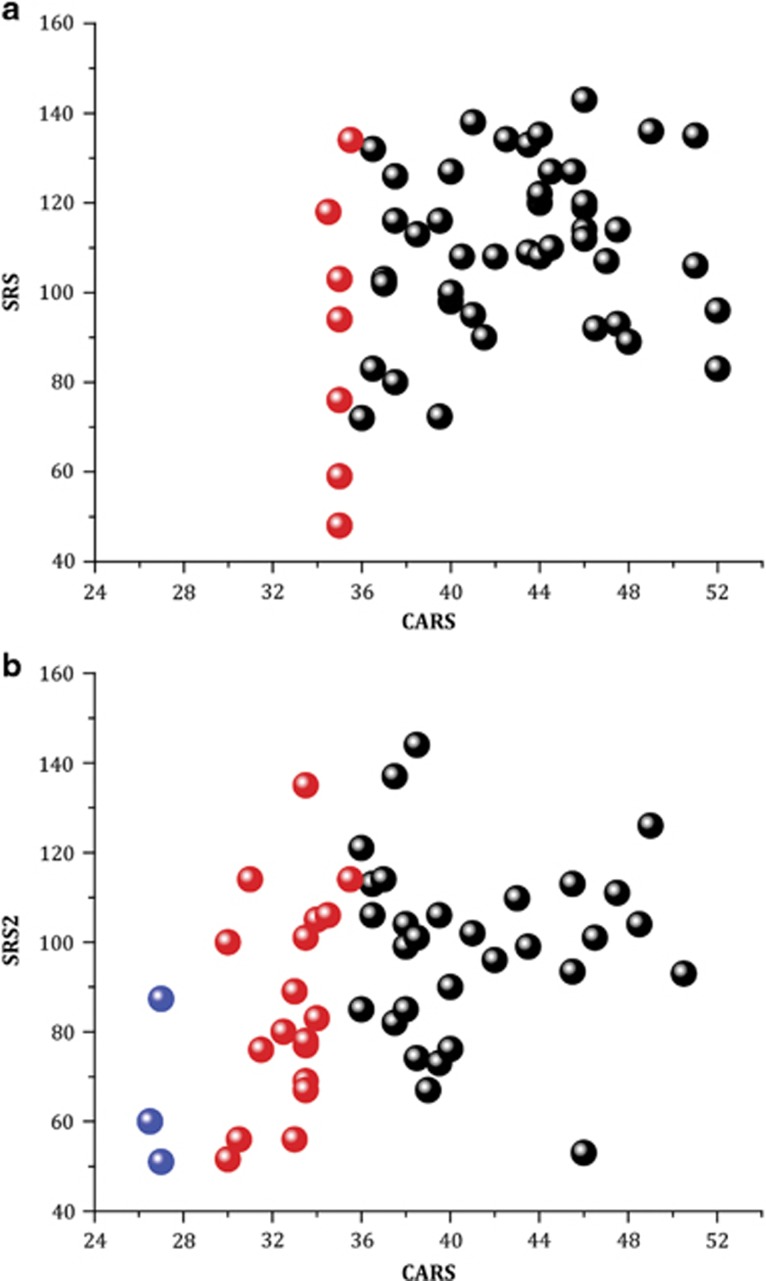
Relationship between behavior scales results for treated patients (completers). (**a**) and (**b**) Relationship between CARS total score and SRS2 score for patients before (**a**) and after (**b**) treatment. CARS <29: mild ASD (blue bubble), 29⩽ CARS <36: moderate ASD (red bubble) and CARS ⩾36: severe ASD (black bubble). ASD, autism spectrum disorder; CARS, Childhood Autism Rating Scale; SRS, Social Responsive Scale.

Owing to the small size of the various subgroups and of the sample, it is difficult to determine quantitative links between the various scales before and after treatment. Such links are, however, qualitatively suggested in Figures 3 and 4. Figure 3 shows the relationship between the CGI-I and the CARS scales. The much-improved patients on the CGI-I scale also exhibited the highest reduction in the CARS scale. Figures 4a and b shows a similar relation between CARS and SRS comparing the alterations of both scales as a function of the severity of ASD determined by cut-off values of <29, 29–36 and >36 points considered as low-, medium- and high-severity ASD, respectively (shown in blue, red and black, respectively). At D0, many children had medium-to-high levels of CARS and none low (<29) scores with high SRS global values. After treatment (D90), many children shifted from severe-to-moderate and from moderate-to-low ASD estimated by CARS and the SRS scores also shifted to the lower part of the ordinate. Therefore, results suggest that the severity of ASD was attenuated in many children in the two scales.

## Discussion

Our first aim in this study was to determine the most favorable dose of bumetanide in the pediatric population. In keeping with earlier studies, our results showed that bumetanide is safe but is associated with adverse events related to diuresis and dehydration. The hypokalemia is clinically manageable with potassium supplementation, dose titration and careful hydration of the patient. This is in keeping with over a decade of experience with loop diuretic drugs. We implemented twice-daily treatment to increase patient exposure to bumetanide given its short lifetime. Results suggest that the frequency and severity of adverse events increased with the dose, whereas there was no clear dose–response relationship for efficacy. The results of this dose-ranging study demonstrated the highest efficacy of bumetanide 2 mg twice a day on both primary and secondary end points. Nevertheless, considering the rate of withdrawals (33.3%), the number (121 TEAEs) and severity of adverse events in dosage group, it will not be retained for further Phase III trials. The dose of 1 mg twice daily appears to be the best compromise between safety and efficacy.

Our observations provide important experience with the medication across the entire pediatric age range.^44, 45, 51, 52^ Although the TEAEs are in accord with well-described earlier reports, they stress the need to control electrolytes and hydration. There was no simple relation between TEAEs and efficacy. Thus, none of the moderate hypokalemic patients (<3 mm l^−1^) were high responder patients with at least 6 points in CARS change from screening to day 90. In the FAS analysis, the highest CARS score decrease was in the 0.5 mg group, whereas the highest frequency in AEs was in the 2.0 mg group. Bumetanide acts on two target tissues (kidney and brain) and on two independent physiological functions. The lack of a direct relationship between efficacy and safety is illustrated by our findings; lack of dose response effect for efficacy but a dose-dependent increase in the frequency and severity of AEs.

Our results suggest that bumetanide is effective in improving ASD-related symptoms across the pediatric age range as assessed by CARS, CGI-I and SRS. The results with CARS are striking when assessing the trial's completers (73 out of 88). Significant effects were also observed with other scales including CGI and the parent-rated SRS total score with an improvement of more than 10 points considered clinically relevant. In keeping with our previous single center trial,^37^ the most consistently altered behavior noted by the parents in the SRS subscales are in social communication and restricted interest. Interestingly, responders were found in all subpopulations, ages and ASD severity according to CARS scores suggesting that the treatment is not restricted to a particular group. The parallel attenuation observed with the three scales stresses the parallelism between the evaluation by parents and child psychiatrist.

Our results confirm the results of smaller trials performed previously.^37, 38, 40^ In the earlier single-site double-blind randomized trial (54 children 3–11 years old), bumetanide (1 mg daily 3 months) improved ASD behaviors as assessed by CARS and CGI. In another trial, children treated with bumetanide and ABA improved more than children with ABA and placebo, by CGI and ABC but not with CARS.^42^ In an open-label study, bumetanide improved visual communication and recognition of emotive figures in eight adolescents with ASD whereas neutral figures were unaffected.^39^ With functional magnetic resonance imaging, emotive but not neutral figures activated important cortical regions after but not before bumetanide treatment, suggesting alterations of threshold activation by relevant images.^39^ Bumetanide restored altered EEGs power spectra in an adolescent with ASD and sensory parameters in another study.^40^ This adolescent had a paradoxical reaction to benzodiazepines that is considered to reflect excitatory actions of GABA and high (Cl^−^)_i_ levels.^5^ Collectively, these studies suggest that bumetanide might offer novel treatment of ASD.

Translating animal research to human treatment is inherently difficult because of the lack of efficient criteria to determine relevant ASD criteria in rodents. In addition, measuring (Cl^−^)_i_ levels in human central neurons is not possible unless surgical resections are performed and thus we do not know whether they are abnormally elevated. Yet, in two ASD animal models, (Cl^−^)_i_ concentrations are elevated in cortical neurons and bumetanide attenuates electrical and behavioral features of the disorder.^34, 35, 36^ Our results suggest that bumetanide known to specifically act on NKCC1 in central neurons attenuated ASD severity. It is not possible to provide a mechanistic explanation as to the attenuation by bumetanide of certain core ASD symptoms but not others. Any conjecture on these complex issues would be at this stage highly speculative. It also however bears stressing that ASD is an early-onset neurodevelopmental disorder, triggered by intrauterine genetic and environmental insults and drugs.^18, 19, 34, 53, 54, 55^ In keeping with the ‘Neuroarcheology' concept,^55^ it is suggested that these early pathogenic events deviate developmental sequences leading to the persistence in the adult brain of immature features including high (Cl^−^)_i_ levels and excitatory GABA actions.^12, 34, 35, 36, 54^ A recent study shows the importance of developmental processes in peripheral integration of sensory information in the pathogenesis of ASD and Rett syndrome.^56^ Therefore, converging experimental and clinical observations suggest that bumetanide might provide a novel therapeutic strategy that improves ASD symptoms without curing it as suggested by the recurrence of symptoms when the treatment is stopped.

There are several important limitations to the present study. The small size of the population and the short observation period limit the determination of the best responders in terms of age, severity and clinical characteristics. It is also not possible at present to claim that bumetanide will act on the entire population of ASD pediatric population as children and adolescents taking other medications and/or having other comorbidities as these were excluded. In spite of the compelling data on high intracellular chloride in animal models, it is impossible to determine these levels in patients with autism and therefore we must rely on the assumptions that this is indeed the mechanism in humans. Nevertheless, the high specificity of bumetanide to brain NKCC1 and kidney NKCC2 chloride importers strongly argues in favor of this assumption. Our small subpopulations excluded an analysis of comorbidity like attention-deficit hyperactivity disorder or depression and its links with efficacy. An additional important limitation concerns the lack of information on treatment response predictors including intelligence quotient, schooling and comorbidity. This hampers the conclusions because of the extreme heterogeneity of ASD, and our future clinical trials will have to incorporate these measures. Finally, the diuretic actions of bumetanide also impact the blinding procedure. To reduce this impact, the psychiatrist was separated from the pediatrician who treated the children and was thus blinded to the treatment. In addition, the convergence of results based on scores made by caregivers and psychiatrists reduces the impact of the diuretic on blinding. Clearly this trial must be viewed as a source of data on the safety and dose-ranging usage of bumetanide and it provides further support to justify a large multisite European Phase III trial.

## Figures and Tables

**Table 1:** Drug-related AEs in ⩾5 patients

	*Bumetanide 0.5 mg b.i.d. (*N*=20)*		*Bumetanide 1 mg b.i.d. (*N*=23)*		*Bumetanide 2 mg b.i.d. (*N*=21)*		*Placebo (*N*=22)*	
	n	N *(%)*	n	N *(%)*	n	N *(%)*	n	N *(%)*
Hypokalemia	6	6 (30%)	20	14 (60.9%)	22	16 (76.2%)	0	0
Diuresis, enuresis, polyuria, pollakiuria	4	2 (10%)	12	8 (34.8%)	13	11 (52.4%)	0	0
Loss of appetite/anorexia	0	0	7	7 (30.4%)	9	9 (42.9%)	0	0
Dehydration	0	0	3	3 (13.0%)	6	6 (28.6%)	0	0
Asthenia	2	2 (10%)	2	2 (8.7%)	4	3 (14.3%)	0	0
Weight loss	0	0	3	3 (13.0%)	3	3 (14.3%)	0	0
Vomiting	0	0	1	1 (4.3%)	5	5 (23.8%)	0	0
Diarrhea/liquid stools	0	0	4	2 (8.7%)	0	0	3	3 (13.6%)
Fatigue	0	0	1	1 (4.3%)	4	4 (19.0%)	0	0
Abdominal pain/belly pain	2	1 (5%)	1	1 (4.3%)	2	2 (9.5%)	0	0
Hyperuricemia	2	1 (5%)	3	3 (13.0%)	1	1 (4.8%)	0	0
Thirst/polydipsia	1	1 (5%)	2	2 (8.7%)	2	2 (9.5%)	0	0

Abbreviations: AEs, adverse events; *n*, number of events; *N*, Number of patients; %, percentage of patients.

The main treatment emergent adverse events (TEAEs) reported in at least five patients in all treatment groups during the randomized phase (incidence reported in % of bumetanide- and placebo-treated patients). Patients with multiple TEAEs within the same category were counted only once towards category total.

**Table 2:** Efficacy outcomes

	*Bumetanide 0.5 mg (*N*=20)*	*Bumetanide 1.0 mg (*N*=23)*	*Bumetanide 2.0 mg (*N*=22)*	*Placebo (*N*=23)*	P*-value*
*CARS (full analysis set)*					
*Screening*					
*N*	20	23	22	23	
Mean (s.d.)	42.45 (4.18)	41.43 (6.01)	41.30 (5.44)	40.41 (4.89)	

*Day 90*					
*N*	20	23	22	23	
Mean (s.d.)	37.48 (5.59)	38.04 (6.13)	38.14 (6.19)	38.78 (4.58)	

*Change*					
Mean (s.d.)	−4.98 (4.33)	−3.09 (3.30)	−3.16 (3.98)	−1.63 (2.34)	
Median [range]	−5.00 [−11.0/3.0]	−2.50 [−10.0/1.0]	−2.00 [−12.0/3.0]	−1.00 [−8.5/2.0]	
					
Kruskal–Wallis test					0.069
*Steel–Dwass pair-wise comparisons*					
0.5 mg b.i.d. vs placebo					0.049
					
*CARS (completers)*					
*Screening*					
*N*	20	23	22	23	
Mean (s.d.)	42.45 (4.18)	41.13 (6.01)	41.30 (5.44)	40.41 (4.89)	

*Day 90*					
*N*	20	19	13	21	
Mean (s.d.)	37.48 (5.59)	37.00 (5.31)	37.73 (7.14)	38.62 (4.60)	

*Change*					
Mean (s.d.)	−4.98 (4.33)	−3.74 (3.28)	−5.35 (3.88)	−1.79 (2.39)	
Median [range]	−5.00 [−11.0/3.0]	−4.00 [−10.0/1.0]	−6.00 [−12.0/3.0]	−1.00 [−8.5/2.0]	
					
Kruskal–Wallis test					0.015
*Steel–Dwass pair-wise comparisons*					
2.0 mg b.i.d. vs placebo					0.017
					
*SRS (total score)*					
*Screening*					
*N*	20	23	22	22	
Mean (s.d.)	113.35 (17.48)	106.42 (23.95)	106.58 (25.09)	112.68 (20.65)	

*Day 90*					
*N*	20	18	13	21	
Mean (s.d.)	100.99 (23.70)	87.96 (19.89)	87.03 (22.03)	109.47 (26.07)	

*Change*					
Mean (s.d.)	−12.36 (23.57)	−13.17 (20.45)	−21.83 (19.78)	−1.55 (20.38)	
Median [range]	−13.00 [−86.0/14.0]	−6.00 [−52.0/18.0]	−27.00 [−47.0/8.0]	3.5 [−68.0/27.0]	
					
Kruskal–Wallis test					0.020
					
*CGI-I*					
*CGI-I at day 90*					
Very much improved	0	0	1 (7.7%)	0	
Much improved	7 (35.0%)	5 (26.3%)	5 (38.5%)	1 (4.8%)	
Minimally improved	7 (35.0%)	10 (52.6%)	7 (53.8%)	10 (47.6%)	
No change	4 (20.0%)	4 (21.1%)	0	9 (42.9%)	
Minimally worse	1 (5.0%)	0	0	1 (4.8%)	
Not assessed/missing	0	4	9	2	
					
Kruskal–Wallis test					0.004

Abbreviations: CARS, Childhood Autism Rating Scale; CGI-I, Clinical Global Impressions Improvement scale; SRS, Social Responsive Scale.

Efficacy outcomes, *N* is number of patients. For CARS and SRS scales, change from screening to day 90. A reduction in CARS and SRS score corresponds to an improvement.

**Table 3:** Proportion of high responders in CARS scores bumetanide- and placebo-treated groups

	*Bumetanide 0.5 mg* *b.i.d. (*N*=20)*	*Bumetanide 1.0 mg* *b.i.d. (*N*=23)*	*Bumetanide 2.0 mg* *b.i.d. (*N*=22)*	*Placebo (*N*=23)*	P*-value* *(chi-square)*	P*-value* *(Fisher's exact test)*
*Responder*						
⩾4	11 (55.0%)	10 (43.5%)	11 (50%)	4 (17.4%)	0.0076	0.0125
⩾6	10 (50.0%)	5 (21.7%)	8 (36.4%)	1 (4.3%)	0.0041	0.0029
⩾8	7 (35.0%)	2 (8.7%)	3 (13.6%)	1 (4.3%)	0.1011	0.1701

Thirty bumetanide- and five placebo-treated showed an attenuation of more than 4 of whom 23 treated and only one placebo showed an amelioration of more than six Childhood Autism Rating Scale (CARS) scores, and 13 of these and only one placebo showed an attenuation of more than 8 points. The differences between placebo- and bumetanide-treated patients having more than 4, 6 or 8 points attenuation are highly significant.

**Table 4:** SRS subscales attenuations by bumetanide

*Restricted interests and* *repetitive behavior*	*Bumetanide* *0.5 mg b.i.d.*	*Bumetanide* *1.0 mg b.i.d.*	*Bumetanide* *2.0 mg b.i.d.*	*Placebo*
*n*	20	18	13	21
Mean value	−1.70	−4.37	−5.78	+0.20
s.d.	5.72	4.37	4.98	4.15
Median value	−1.50	−3.50	−4.00	0.00

*Social communication*	*Bumetanide* *0.5 mg* *b.i.d.*	*Bumetanide* *1.0 mg b.i.d.*	*Bumetanide* *2.0 mg b.i.d.*	*Placebo*
*n*	20	18	12	21
Mean value	−4.38	−3.09	−9.20	−1.25
s.d.	8.84	8.95	7.61	6.72
Median value	−4.5	−4.5	−11.0	−0.5

Statistically significant attenuations of Social Responsive Scale (SRS) subscales by bumetanide. The difference between treated and placebo were statistically different in social communication (Kuskaal–Wallis test *P*=0.03) and restricted interest and repetitive behavior (Kuskaal–Wallis test *P*=0.002).
