# Effects of bumetanide on neurobehavioral function in children and adolescents with autism spectrum disorders

**DOI:** 10.1038/tp.2017.101

**Published:** 2017-05-09

**Authors:** E Lemonnier, N Villeneuve, S Sonie, S Serret, A Rosier, M Roue, P Brosset, M Viellard, D Bernoux, S Rondeau, S Thummler, D Ravel, Y Ben-Ari

**Correction to:** *Translational Psychiatry* (2017) **7**, e1056; doi:10.1038/tp.2017.10; published online 14 March 2017

The ninth author’s affiliation was presented incorrectly in the published paper. Additionally, only the first affiliation should have included CHU Hôpital Le Cluzeau. The correct affiliations appear below:

E Lemonnier^1^, N Villeneuve^2^, S Sonie^3^, S Serret^4^, A Rosier^5^, M Roue^6^, P Brosset^1^, M Viellard^2^, D Bernoux^7^, S Rondeau^5^, S Thummler^4^, D Ravel^8^ and Y Ben-Ari^8,9^

^1^CHU Hôpital Le Cluzeau, CHU Limoges, Limoges, France

^2^Hôpital Sainte Marguerite, Marseille, France

^3^CHU le Vinatier, Bron, France

^4^CHU Lenval, Nice, France

^5^CHU Le Rouvray, Sotteville les Rouen, France

^6^CHRU Brest, Brest, France

^7^EPICIME-CIC 1407 de Lyon, Hospices Civils de Lyon, Inserm, Bron, France

^8^Neurochlore Research Team, Marseille, France

^9^Neurochlore Research Team, C/O INMED, INSERM Unité 901, Aix Marseille Université, Marseille, France

