# Supplementary material for: Effects of bumetanide on neurobehavioral function in children and adolescents with autism spectrum disorders
Source: Transl Psychiatry. 2017 Mar 14;7(3):e1056–. doi: 10.1038/tp.2017.10 (PMC5416661; doi:10.1038/tp.2017.10)
Supplement: Supplementary Information [file tp201710x1.pdf]

Pharmacokinetic analysis

Blood sampling in children who participate in medical research is always an issue of concern to parents, nurses, and clinicians for obvious ethical reasons notably when dealing with children with ASD. To prevent pain and stress related to blood sampling in children, in accordance with EMA experts, we decided to perform a Pharmacokinetic Population (POP-PK) approach requiring fewer blood samples per patient. The objective was to demonstrate that the PK profile of Bumetanide in pediatric population was comparable to that reported in adults. An optimized blood sampling schedule limiting the number of blood samples collected in each patient was developed taking into account the constraints of the study population in terms of age and disease characteristics. A review of the PK data collected in literature led to the generation of a PK blood-sampling schedule with only 6 blood samples drawn over a 3 months period for the children (aged ≥2 to <12 years) and 8 samples drawn over the same period for adolescents (aged ≥12 to ≤18 years). Determination of bumetanide in human plasma was performed with a validated LC/MS-MS method with a limit of quantification of 1ng/mL. Several pharmacokinetic studies have shown that bumetanide, administered orally is eliminated rapidly in humans, with a half-life between 1 and 1½ hours. In our study half-life ranged from 0.37 h to 1.4 hour.

Treatment Emergent Adverse Events (TEAEs)

TEAEs were defined as "any adverse drug experience occurring at any dose that results in *Inpatient hospitalization* ». Ten patients having received Bumetanide experienced a moderate hypokalemia (K below 3.0 mM). For 2 of them, a SAE was reported due to hospitalization. For both cases, parents were concerned by the bad clinical state of their children mainly due to dehydration. They went directly to the emergency department of a nearby hospital. Blood analyses revealed a potassium level below 3 mM considered as a significant event. They received K intravenously or orally and improved rapidly. Other hypokalemia TEAEs were managed directly by the pediatricians of the clinical center involved in the study. They were reported as AEs.

There is no straightforward relationship between age and adverse events. Although Bumetanide is not presently recommended for the treatment of edema in children below 12 years of age, it has been shown that it is a potent diuretics in paediatrics (Wells

et al, 1992 and Marshall et al., 1998)). When the age range was split into two subgroups 2-8 and 9-18 year-old, the number of patients experiencing clinically significant mild and moderate hypokalemia was roughly the same in both groups (**supplemental Table 2)**. For the second most frequent AE including diuresis, enuresis, polyurea, pollakiuria, a total of 23 patients experienced at least one of these AES. The distribution amongst the age groups was also homogeneous.

Post-hoc parametric analyses:

The change from screening to D90 in CARS was also analyzed using a general linear model (normal distribution) with screening value as covariate in the FAS. In order to take into account the multiplicity, Dunnett's multiple comparison procedure was used. In the FAS, the mean values for CARS across all Bumetanide treated groups decreased more than in the placebo group. The Dunnett's multiple comparison showed that the difference between Bumetanide 0.5 mg and placebo was statistically significant (p = 0.015).

Supplemental Figure legends

Supplemental Figure 1: Population pharmacokinetic model and median observed profiles for 0.5, 1.0 and 2.0 mg BID treated groups. A. Dose level 0.5 mg BID, Day 90. B. Dose level 1.0 mg BID, Day 90. C. Dose level 2.0 mg BID, Day 90. Blue aera: 90% observation quantile band, Red line: Median observed profile, Black line: Median population model prediction.

Supplemental Figure 2: Mean plot of blood potassium (safety set). Blue line: Bumetanide 0.5 mg BID, Red line: Bumetanide 1.0 mg BID, Black line: Bumetanide 2.0 mg BID, Green line: Placebo. SEM values.

Supplemental Figure 3: time of occurrence of hypokalemia and dose dependence**. A:** number of patients experiencing hypokalemia and time of occurrence. Note that this was conspicuous the first week of treatment. **B:** dose dependence, note that the hypokalemia was more frequent with the 01 and 2mg than the 0.5mg dose of bumetanide. It also occurred more frequently during the first week.

Supplemental figure 4: CARS data fit a normal curve. **Top**: Study of response normality, fitting density plot by treatment group. Study of resident normality; fitting density plots. **Bottom**: study of resident normality; Normal probability plot.

Supplemental Figure 5: SRS data fit a normal curve. **Top**: fitting density plot by treatment group. **Bottom**: study of resident normality; Normal probability plot.

Supplemental Table legends

S**upplemental Table 1:** Demography and initial diagnosis for 88 study participants randomly assigned to receive Bumetanide or placebo.

Supplemental table 2: Frequency of moderate hypokalemia was comparable when patients are split into two age subgroups 2-18 and 9-18 years old. N= Number of patients.

Supplemental Table 3: Analysis of covariance with CARS screening value as covariate and considering *the LOCF meth*od for handling missing data at D90 (*).
(1) Estimate (Standard Error) of the difference between adjusted treatment group means : Bumetanide dose minus Placebo.
(2) Two-sided 95% Adjusted Confidence Interval of the estimate (with Dunnett-based adjustment).
(3) Two-sided Adjusted p-value (adjustment according to the procedure of Dunnett-Hsu for multiplicity adjustment).

Supplemental Table 4: Analysis of covariance with CARS screening value as covariate and considering *the multiple imputation* method for handling missing data at D90.
(1) Estimate (Standard Error) of the difference between adjusted treatment group means : Bumetanide dose minus Placebo.
(2) Two-sided 95% Adjusted Confidence Interval of the estimate (with Dunnett-based adjustment)
(3) Two-sided Adjusted p-value (adjustement according to the procedure of Dunnett-Hsu for multiplicity adjustment).

Supplemental Table 5 : Analysis of covariance with SRS screening value as covariate and considering *the LOCF method* for handling missing data at D90(*). (1) Estimate (Standard Error) of the difference between adjusted treatment group means : Bumetanide dose minus Placebo. (2) Two-sided 95% Adjusted Confidence Interval of the estimate (with Dunnett-based adjustment). (3) Two-sided Adjusted p-value (adjustement according to the procedure of Dunnett-Hsu for multiplicity adjustment).

Supplemental Table 6 : Descriptive statistics are done based on available data (before imputation). Analysis of covariance with SRS screening value as covariate and considering *the multiple imputation method* for handling missing data at D90.
(1) Estimate (Standard Error) of the difference between adjusted treatment group means : Bumetanide dose minus Placebo.
(2) Two-sided 95% Adjusted Confidence Interval of the estimate (with Dunnett-based adjustment).
(3) Two-sided Adjusted p-value (adjustement according to the procedure of Dunnett-Hsu for multiplicity adjustment).

Supplemental table 1

	Bumetanide 0.5 mg BID	Bumetanide 1.0 mg BID	Bumetanide 2.0 mg BID	Placebo	All groups
**AGE (YEARS)**					
Mean (SD)	7.80 (4.15)	7.87 (4.58)	8.45 (4.57)	8.87 (4.96)	8.26 (4.53)
Median [Range]	7.00 [2.0-15.0]	7.00 [2.0-17.0]	7.00 [2.0-17.0]	8.00 [2.0-17.0]	7.00 [2.0-17.0]
**SEX**					
Male	16 (80.0%)	22 (95.7%)	21 (95.5%)	19 (82.6%)	78 (88.6%)
Female	4 (20.0%)	1 (4.3%)	1 (4.5%)	4 (17.4%)	10 (11.4%)
**INITIAL DIAGNOSIS**					
F84.0 (Childhood Autism)	18 (90.0%)	23 (100%)	21 (95.5%)	20 (87.0%)	82 (93.2%)
F84.5 (Asperger Syndrome)	2 (10.0%)	0	1 (4.5%)	3 (13.0%)	6 (6.8%)

Supplemental Table 2

hypokalemia during the study		Bumetanide 0.5 mg BID (N=20)	Bumetanide 1 mg BID (N=23)	Bumetanide 2 mg BID (N=21)	Placebo (N=22)	Total (N=86)
Age 2-8 years	Moderate (<3 mEq/L)	0	3 (23.1%)	3 (27.3%)	0	6 (12.8%)
	Mild (3 to <3.5 mEq/L)	3 (27.3%)	6 (46.2%)	4 (36.4%)	0	13 (27.7%)
	No hypokalemia	8 (72.7%)	4 (30.8%)	4 (36.4%)	12 (100.0%)	28 (59.6%)
Age 9-18 years	Moderate (<3 mEq/L)	0	4 (40.0%)	0	0	4 (10.3%)
	Mild (3 to <3.5 mEq/L)	4 (44.4%)	2 (20.0%)	8 (80.0%)	0	14 (35.9%)
	No hypokalemia	5 (55.6%)	4 (40.0%)	2 (20.0%)	10 (100.0%)	21 (53.8%)

Supplemental Table 3

		Bumetanide 0.5 mg bid (N=20)	Bumetanide 1.0 mg bid (N=23)	Bumetanide 2.0 mg bid (N=22)	Placebo (N=23)
Descriptive Statistics					
SCREENING	N	20	23	22	23
	Mean ± SD	42.45 ± 4.18	41.13 ± 6.01	41.30 ± 5.44	40.41 ± 4.89
	Median	43.75	39.50	40.75	39.00
	Q1 ; Q3	39.00 ; 46.00	36.00 ; 46.00	35.50 ; 46.00	36.50 ; 43.50
	Min ; Max	35.0 ; 49.0	34.5 ; 52.0	34.5 ; 52.0	34.5 ; 51.5
D90(*)	N	20	23	22	23
	Mean ± SD	37.48 ± 5.59	38.04 ± 6.13	38.14 ± 6.19	38.78 ± 4.58
	Median	37.75	36.00	37.00	37.50
	Q1 ; Q3	34.00 ; 39.00	33.50 ; 41.00	33.50 ; 41.50	35.50 ; 42.00
	Min ; Max	26.5 ; 49.0	27.0 ; 51.5	27.0 ; 50.5	30.0 ; 47.5
D90(*) - SCREENING	N	20	23	22	23
	Mean ± SD	-4.98 ± 4.33	-3.09 ± 3.30	-3.16 ± 3.98	-1.63 ± 2.34
	Median	-5.00	-2.50	-2.00	-1.00
	Q1 ; Q3	-9.00 ; -1.25	-5.00 ; 0.00	-6.00 ; 0.00	-3.50 ; 0.00
	Min ; Max	-11.0 ; 3.0	-10.0 ; 1.0	-12.0 ; 3.0	-8.5 ; 2.0
Statistical analysis					
D90(*) - SCREENING	E (SE) (1)	-3.061 (1.074)	-1.356 (1.027)	-1.406 (1.039)	
	95% CI (2)	[-5.633 ; -0.488]	[-3.816 ; 1.104]	[-3.895 ; 1.084]	
	p-value (3)	0.015	0.416	0.397	

Supplemental Table 4

		Bumetanide 0.5 mg bid (N=20)	Bumetanide 1.0 mg bid (N=23)	Bumetanide 2.0 mg bid (N=22)	Placebo (N=23)
Descriptive Statistics					
SCREENING	N	20	23	22	23
	Mean ± SD	42.45 ± 4.18	41.13 ± 6.01	41.30 ± 5.44	40.41 ± 4.89
	Median	43.75	39.50	40.75	39.00
	Q1 ; Q3	39.00 ; 46.00	36.00 ; 46.00	35.50 ; 46.00	36.50 ; 43.50
	Min ; Max	35.0 ; 49.0	34.5 ; 52.0	34.5 ; 52.0	34.5 ; 51.5
D90	N	20	19	13	21
	Mean ± SD	37.48 ± 5.59	37.00 ± 5.31	37.73 ± 7.14	38.62 ± 4.60
	Median	37.75	36.00	38.00	37.50
	Q1 ; Q3	34.00 ; 39.00	33.50 ; 39.50	32.50 ; 43.00	35.50 ; 41.50
	Min ; Max	26.5 ; 49.0	27.0 ; 48.5	27.0 ; 50.5	30.0 ; 47.5
D90 - SCREENING	N	20	19	13	21
	Mean ± SD	-4.98 ± 4.33	-3.74 ± 3.28	-5.35 ± 3.88	-1.79 ± 2.39
	Median	-5.00	-4.00	-6.00	-1.00
	Q1 ; Q3	-9.00 ; -1.25	-7.00 ; -1.00	-6.50 ; -5.50	-3.50 ; 0.00
	Min ; Max	-11.0 ; 3.0	-10.0 ; 1.0	-12.0 ; 3.0	-8.5 ; 2.0
Statistical analysis					
Multiple imputation	E (SE) (1)	-3.018 (1.140)	-1.735 (1.092)	-3.964 (1.666)	
	95% CI (2)	[-5.750 ; -0.287]	[-4.350 ; 0.880]	[-7.955 ; 0.027]	
	p-value (3)	0.026	0.271	0.052	

Supplemental Table 5

		Bumetanide 0.5 mg bid (N=20)	Bumetanide 1.0 mg bid (N=23)	Bumetanide 2.0 mg bid (N=22)	Placebo (N= 23)
Descriptive Statistics					
SCREENING	N	20	23	22	22
	Mean ± SD	113.35 ± 17.48	106.42 ± 23.95	106.58 ± 25.09	112.68 ± 20.65
	Median	113.00	109.00	111.50	115.50
	Q1 ; Q3	98.50 ; 129.50	96.00 ; 125.00	93.00 ; 127.00	101.00 ; 128.00
	Min ; Max	83.0 ; 143.0	48.0 ; 141.0	54.5 ; 137.0	71.0 ; 156.0
D90(*)	N	20	23	22	23
	Mean ± SD	100.99 ± 23.70	96.11 ± 24.33	93.68 ± 25.92	111.34 ± 25.62
	Median	105.50	101.00	93.22	113.00
	Q1 ; Q3	89.50 ; 113.50	77.00 ; 114.00	76.17 ; 109.75	101.00 ; 130.00
	Min ; Max	51.6 ; 144.0	56.0 ; 141.0	51.0 ; 137.0	62.0 ; 163.0
D90(*) - SCREENING	N	20	23	22	22
	Mean ± SD	-12.36 ± 23.57	-10.31 ± 18.81	-12.90 ± 18.56	-1.41 ± 19.39
	Median	-13.00	-3.00	0.00	2.50
	Q1 ; Q3	-25.50 ; 6.50	-18.00 ; 0.00	-30.00 ; 0.00	-2.00 ; 7.00
	Min ; Max	-86.4 ; 14.0	-52.2 ; 18.0	-47.0 ; 8.0	-68.0 ; 27.0
Statistical analysis					
D90(*) - SCREENING	E (SE) (1)	-10.771 (5.962)	-10.581 (5.786)	-13.130 (5.847)	
	95% CI (2)	[-25.046 ; 3.504]	[-24.434 ; 3.272]	[-27.130 ; 0.871]	
	p-value (3)	0.181	0.173	0.071	

Supplemental Table 6

		Bumetanide 0.5 mg bid (N=20)	Bumetanide 1.0 mg bid (N=23)	Bumetanide 2.0 mg bid (N=22)	Placebo (N=23)
Descriptive Statistics					
SCREENING	N	20	23	22	22
	Mean ± SD	113.35 ± 17.48	106.42 ± 23.95	106.58 ± 25.09	112.68 ± 20.65
	Median	113.00	109.00	111.50	115.50
	Q1 ; Q3	98.50 ; 129.50	96.00 ; 125.00	93.00 ; 127.00	101.00 ; 128.00
	Min ; Max	83.0 ; 143.0	48.0 ; 141.0	54.5 ; 137.0	71.0 ; 156.0
D90	N	20	18	13	21
	Mean ± SD	100.99 ± 23.70	87.96 ± 19.89	87.03 ± 22.03	109.47 ± 26.07
	Median	105.50	86.17	89.00	112.00
	Q1 ; Q3	89.50 ; 113.50	73.00 ; 102.00	76.17 ; 93.44	101.00 ; 128.00
	Min ; Max	51.6 ; 144.0	56.0 ; 121.0	51.0 ; 135.0	62.0 ; 163.0
D90 - SCREENING	N	20	18	13	20
	Mean ± SD	-12.36 ± 23.57	-13.17 ± 20.45	-21.83 ± 19.78	-1.55 ± 20.38
	Median	-13.00	-6.00	-27.00	3.50
	Q1 ; Q3	-25.50 ; 6.50	-30.66 ; 0.66	-37.00 ; 0.00	-5.00 ; 7.55
	Min ; Max	-86.4 ; 14.0	-52.2 ; 18.0	-47.0 ; 8.0	-68.0 ; 27.0
Statistical analysis					
Multiple imputation	E (SE) (1)	-10.621 (6.595)	-17.287 (6.828)	-22.691 (7.407)	
	95% CI (2)	[-26.417 ; 5.175]	[-33.640 ; -0.934]	[-40.431 ; -4.950]	
	p-value (3)	0.261	0.036	0.008	
