# Supplementary material for: Effects of bumetanide on neurobehavioral function in children and adolescents with autism spectrum disorders
Source: Transl Psychiatry. 2017 Mar 14;7(3):e1056–. doi: 10.1038/tp.2017.10 (PMC5416661; doi:10.1038/tp.2017.10)
Supplement: Supplementary Figure 1 [file tp201710x2.pdf]

A

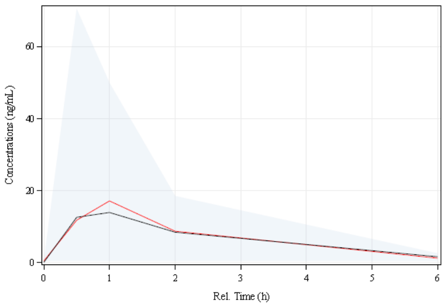

B

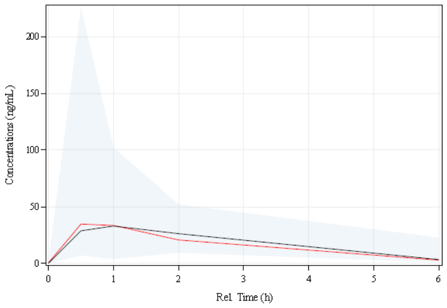

C

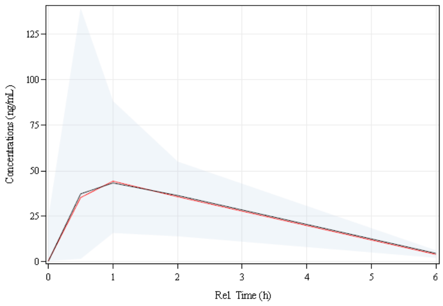

Supplemental Figure 1: Population pharmacokinetic model and median observed profiles for 0.5, 1.0 and 2.0 mg BID treated groups. A. Dose level 0.5 mg BID, Day 90. B. Dose level 1.0 mg BID, Day 90. C. Dose level 2.0 mg BID, Day 90. Blue aera: 90% observation quantile band, Red line: Median observed profile, Black line: Median population model prediction.
