# Supplementary figures and images for: Effects of bumetanide on neurobehavioral function in children and adolescents with autism spectrum disorders
Source: Transl Psychiatry. 2017 Mar 14;7(3):e1056–. doi: 10.1038/tp.2017.10 (PMC5416661; doi:10.1038/tp.2017.10)

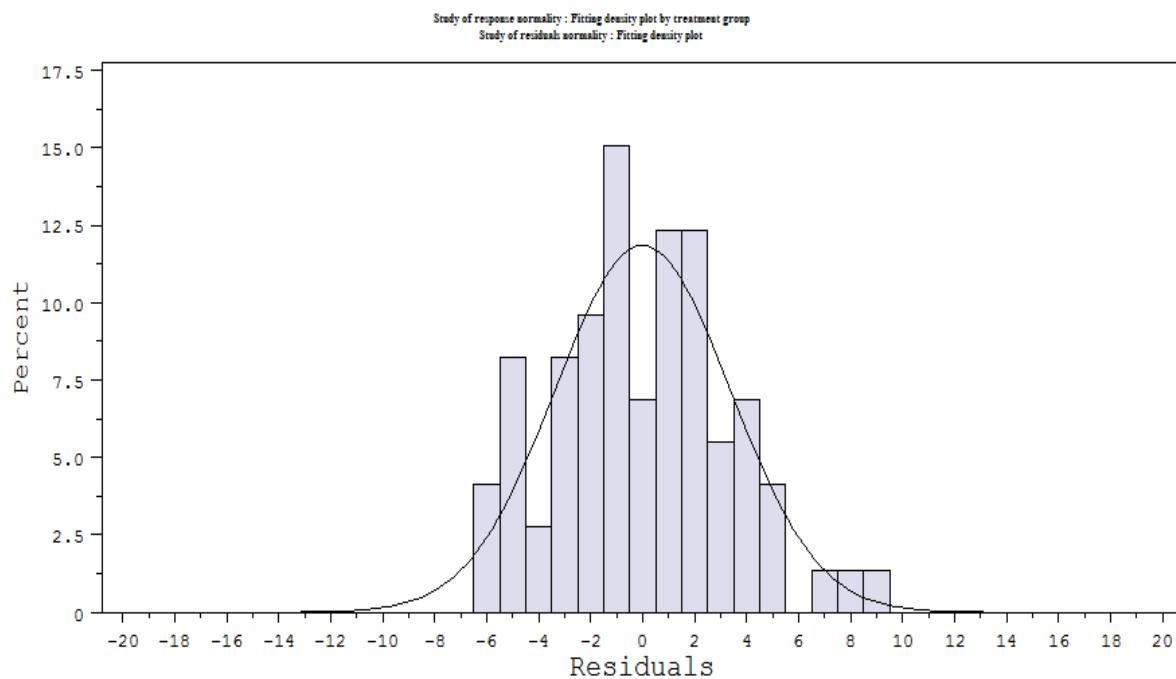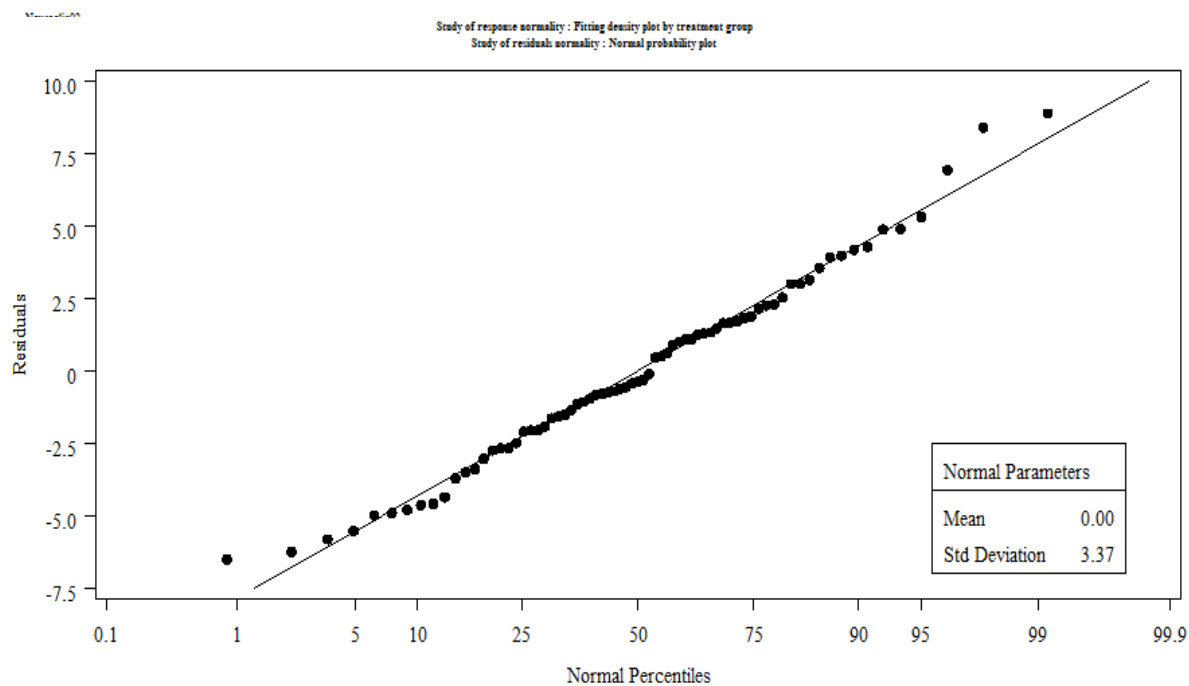

Supplement: Supplementary Figure 4 [file tp201710x3.pdf]

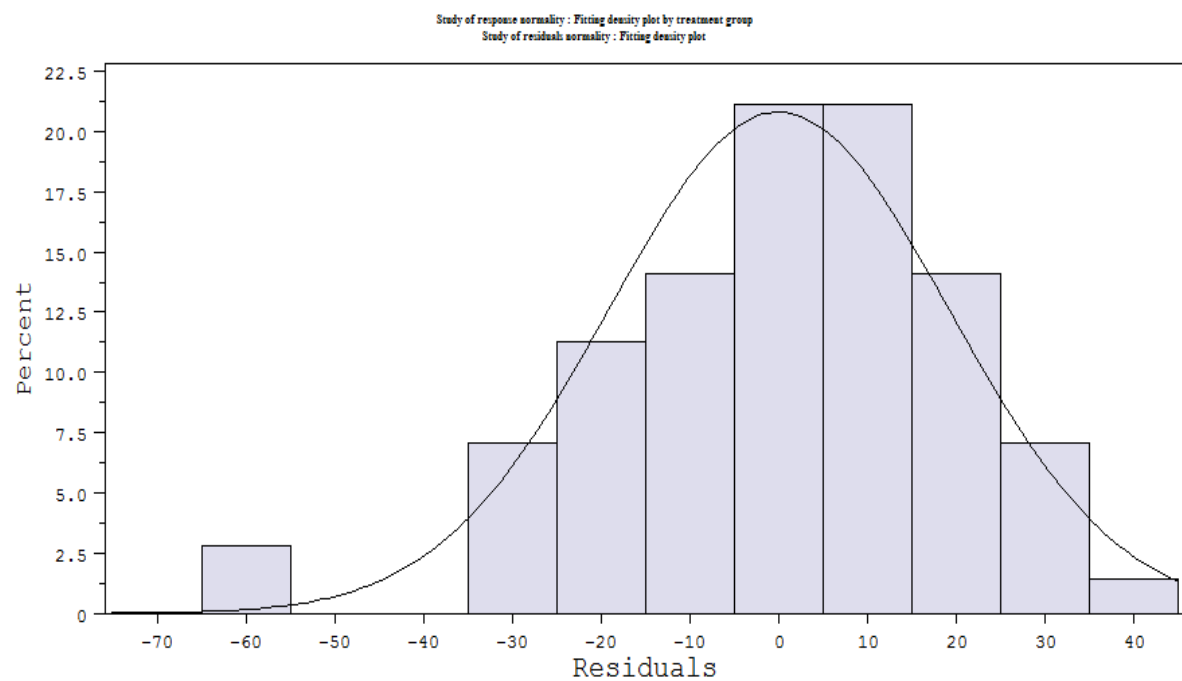

Neuroclin02

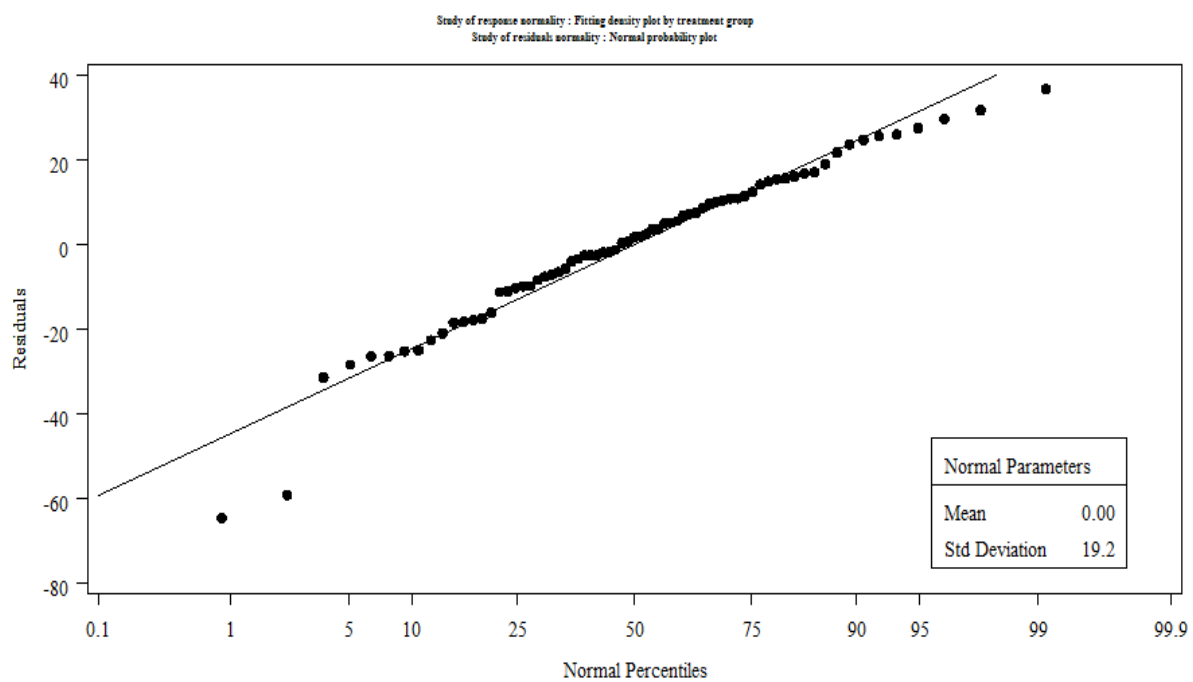

Neuroclin02

Supplement: Supplementary Figure 5 [file tp201710x4.pdf]

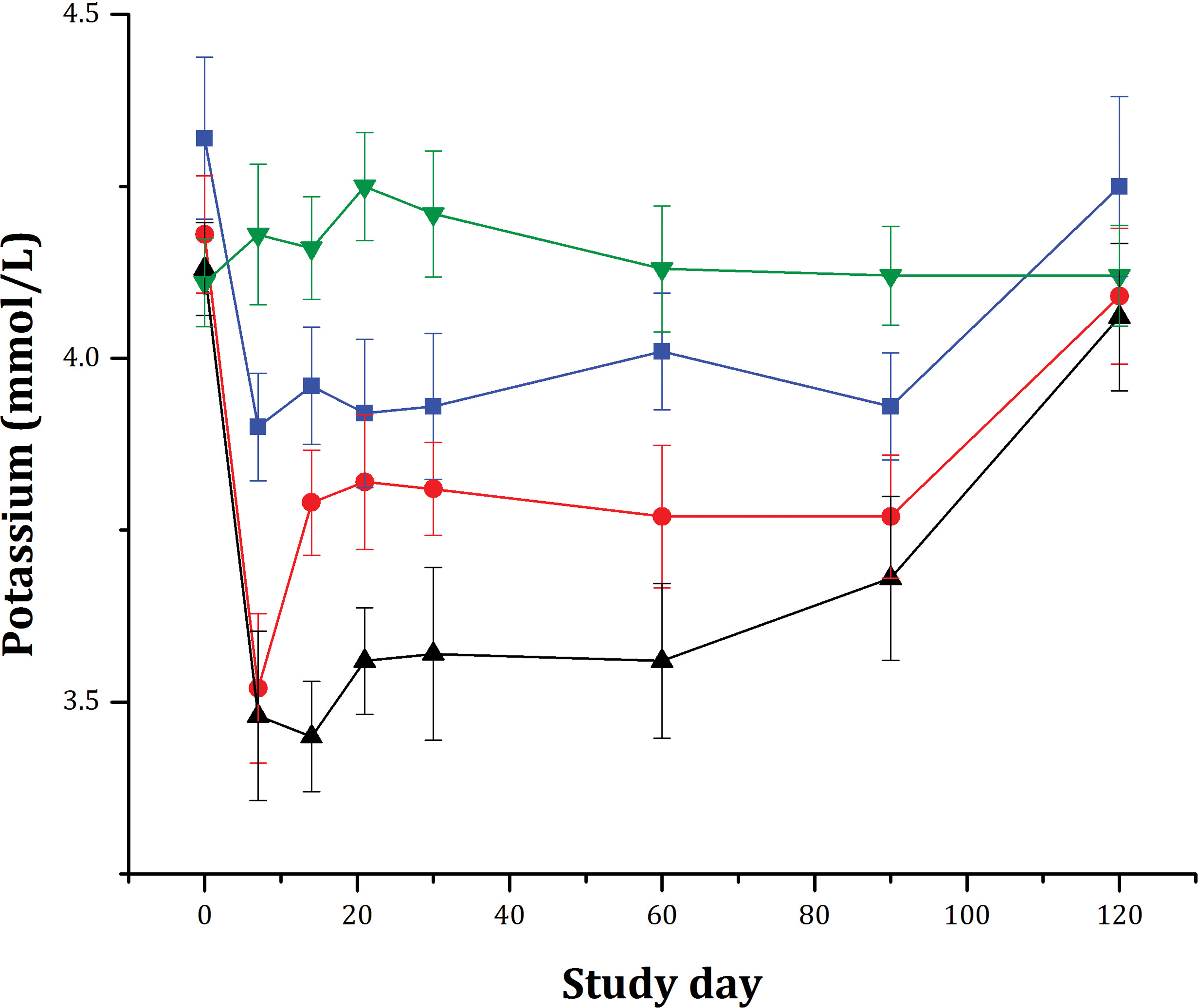

Supplement: Supplementary Figure 2 [file tp201710x5.tif]

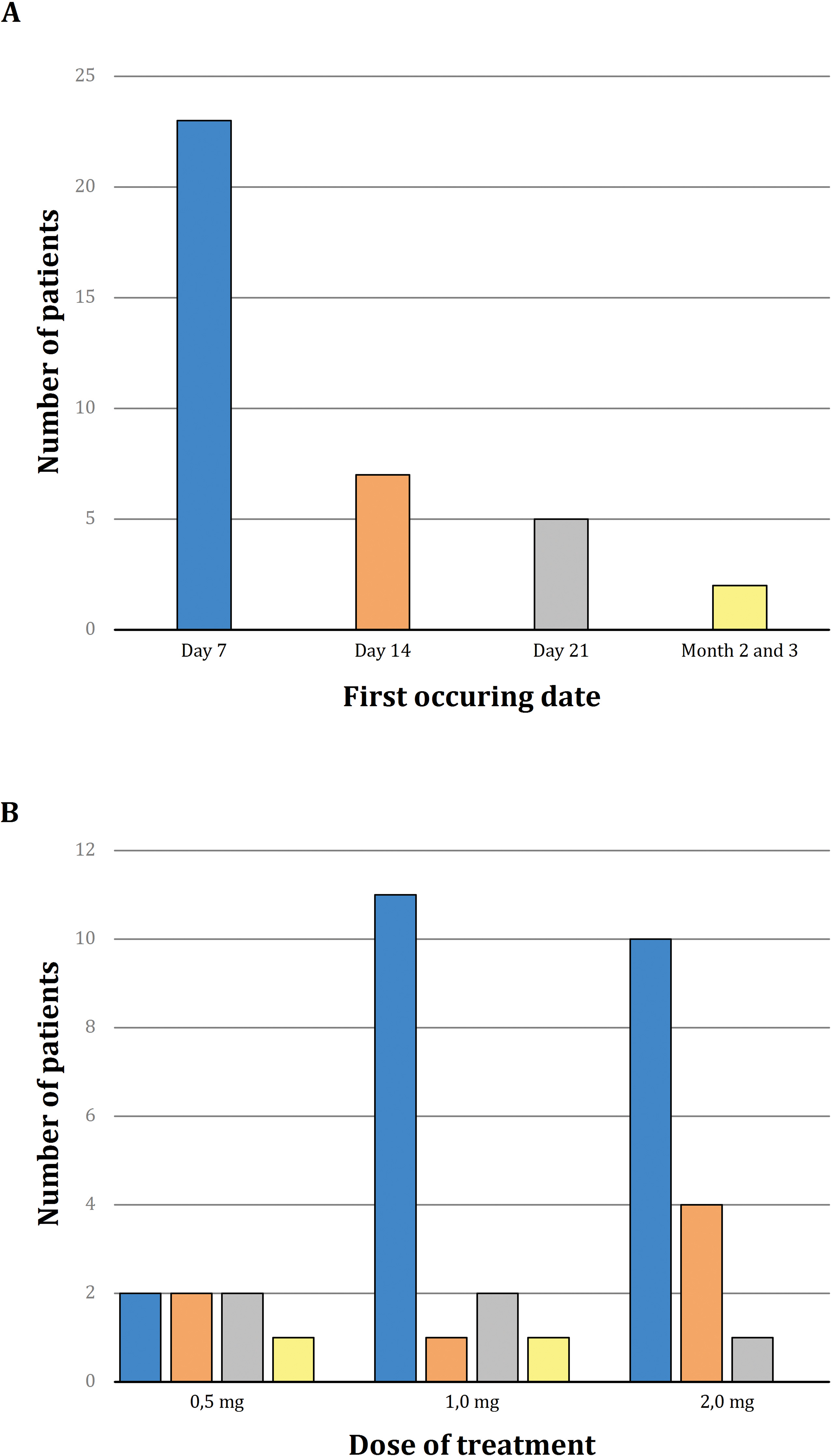

Supplement: Supplementary Figure 3 [file tp201710x6.tif]
